# Extracellular DNA release from the genome-reduced pathogen *Mycoplasma hyopneumoniae* is essential for biofilm formation on abiotic surfaces

**DOI:** 10.1038/s41598-018-28678-2

**Published:** 2018-07-10

**Authors:** Benjamin B. A. Raymond, Cheryl Jenkins, Lynne Turnbull, Cynthia B. Whitchurch, Steven P. Djordjevic

**Affiliations:** 1 The ithree Institute, University of Technology Sydney, Ultimo, NSW 2007 Australia; 2 NSW Department of Primary Industries, Elizabeth Macarthur Agricultural Institute, PMB 8, Camden, NSW Australia

## Abstract

*Mycoplasma hyopneumoniae* is an economically devastating, globally disseminated pathogen that can maintain a chronic infectious state within its host, swine. Here, we depict the events underpinning *M*. *hyopneumoniae* biofilm formation on an abiotic surface and demonstrate for the first time, biofilms forming on porcine epithelial cell monolayers and in the lungs of pigs, experimentally infected with *M*. *hyopneumoniae*. Nuclease treatment prevents biofilms forming on glass but not on porcine epithelial cells indicating that extracellular DNA (eDNA), which localises at the base of biofilms, is critical in the formation of these structures on abiotic surfaces. Subpopulations of *M*. *hyopneumoniae* cells, denoted by their ability to take up the dye TOTO-1 and release eDNA, were identified. A visually distinct sub-population of pleomorphic cells, that we refer to here as large cell variants (LCVs), rapidly transition from phase dark to translucent “ghost” cells. The translucent cells accumulate the membrane-impermeable dye TOTO-1, forming readily discernible membrane breaches immediately prior to lysis and the possible release of eDNA and other intracellular content (public goods) into the extracellular environment. Our novel observations expand knowledge of the lifestyles adopted by this wall-less, genome-reduced pathogen and provide further insights to its survival within farm environments and swine.

## Introduction

Mycoplasmas evolved by a process of degenerative evolution, shedding genes for the biosynthesis of a cell wall, nucleic acids, amino acids, and the tricarboxylic acid cycle. As such mycoplasmas form intimate associations with their respective hosts to sequester essential metabolites needed for growth. Mycoplasmas typically colonise and infect a restricted range of hosts and are not known to proliferate in the environment for extended periods, but can survive in mucosal secretions and travel considerable distances on wind currents^1,2^. The inability of mycoplasmas to synthesize a cell wall is thought to render them vulnerable to desiccation, osmotic stress, and to membrane attacks by products of both the innate and adaptive immune responses during infection. Nonetheless, mycoplasmas are remarkably successful parasitic bacteria that typically induce a chronic infectious state in their respective hosts and cause significant economic losses to both animal and plant industries.

The mycoplasmas are phylogenetically-related to the low G + C Firmicutes and have been assigned to five major and distinct clades based on whole genome comparisons^3^. The precise mechanisms employed by *Mycoplasma* spp. and other bacterial pathogens to achieve a persistent infectious state are not well understood but their ability to form biofilms is considered significant^4–9^. Several mycoplasma species are known to form biofilms^5,8–11^ including representatives associated with Clades II, IV, and V. Many biofilm-forming species synthesize a cytoskeletal organelle and reside in Clade V. The organelle contributes to cell polarity and motility, while focussing critical adhesins to the tip of the organelle for attachment^12^; however, several species that form well-defined biofilms are devoid of an attachment organelle^4,5,10^. The chemical composition of the extracellular matrices that bind mycoplasma biofilms are not well defined, but are known to consist of typical extrapolymeric substances such as proteins, lipids, nucleic acids, and polysaccharides^6,7^. Extracellular DNA (eDNA) is a prominent component of many bacterial biofilms^13,14^, yet no study has investigated a role for eDNA in mycoplasma biofilm formation to date.

*M*. *hyopneumoniae* belongs to Clade III^3^ and is considered to be a surface pathogen that selectively adheres to receptors on ciliated respiratory epithelium^15^. Disruption of the mucociliary escalator via ciliostasis, cilial loss, and epithelial cell death are all hallmarks of infection caused by *M*. *hyopneumoniae*^16–18^. These pathological effects create a favourable microenvironment for infection by secondary bacterial and viral pathogens^17,19,20^. Detailed ultrastructural studies depicting *M*. *hyopneumoniae* interacting with porcine ciliated epithelium show what appear to be microcolonies, attaching along the length of cilia inducing cilial clumping, and splitting, but rarely to the surface of the epithelial cells^15,16,18,21,22^. However, these microcolonies have never been referred to as “biofilms” in the literature. Previous studies have described the presence of so-called “persister cells” within the trachea of swine, described as slow growing or non-dividing *M*. *hyopneumoniae* cells that display a higher threshold of antibiotic susceptibility when compared with exponentially growing cells^23^. It is generally accepted that the individual cells that comprise microbial biofilms are not themselves resistant to antibiotics, but it is the combination of the extrapolymeric matrix and persister cells that contribute to the overall tolerance^24,25^. It has been shown that *M*. *hyopneumoniae* cells recovered from the trachea of pigs 24 h after treatment with a clinically-effective dose of marbofloxacin remain susceptible to marbofloxacin^23^, consistent with the “persister cell” hypothesis. Persister cells are thought to make up a significant proportion of cells within bacterial biofilms and potentially play an important role during recovery of the pathogen after antibiotic treatment and host immune attack^24^. A recent study showed limited evidence of *M*. *hyopneumoniae* forming biofilms on abiotic surfaces that are significantly more resistant to antibiotics than planktonic cells^26^. Additionally, *M*. *hyopneumoniae* has recently been demonstrated to survive for up to 8 days on abiotic surfaces at 4 °C^27^. However, it is not known whether biofilms play a role in this survival.

Here we show that not only does *M*. *hyopneumoniae* form biofilms on abiotic surfaces, but that eDNA is crucial for their formation. We also show, for the first time, *M*. *hyopneumoniae* forming biofilms on the surface of porcine epithelial cell monolayers and on epithelial cell surfaces in the porcine respiratory tract after experimental infection. We provide evidence for morphological variants of *M*. *hyopneumoniae*, referred to here as Large Cell Variants (LCVs) and their role in biofilm formation. Specifically, we characterise the structural and behavioural features of LCVs and show that their inherent instability may contribute to the release of eDNA, essential for forming biofilms on abiotic surfaces.

## Results

### *M. hyopneumoniae* forms biofilms on PK-15 monolayers

PK-15 cells have been used as a model cell-line to study adherence of *M*. *hyopneumoniae*^28–33^. As biofilm formation on abiotic surfaces has been previously demonstrated in *M*. *hyopneumoniae*^26^, we sought to determine if this pathogen can form biofilms on the surface of PK-15 monolayers. This was achieved by allowing the lab adapted *M*. *hyopneumoniae* strain J to adhere to PK-15 cells for 16 h, followed by labelling *M*. *hyopneumoniae* cells with F2_P94-J_ antisera that detects P97 adhesins on the extracellular membrane surface, and Phalloidin CF 568 to visualise filamentous actin within host cells. Biofilms and microcolonies formed rapidly (within 16 h) on PK-15 cells indicating that the surface of PK-15 cells is conducive to initiating and maintaining biofilms formed by *M*. *hyopneumoniae* (Supplementary Fig. S1). Additionally, biofilms that formed on PK-15 cell monolayers mimicked the sizes of those that formed on glass as presented later (Supplementary Fig. S1). The biofilm structures imaged using scanning electron microscopy (SEM) showed complexities about biofilm structure that were not evident in confocal microscopy (Supplementary Fig. S1), namely that there were pleomorphic *M*. *hyopneumoniae* cells that comprised the biofilms (discussed below).

### *M. hyopneumoniae* forms biofilms in the swine respiratory tract

**Figure 1:**
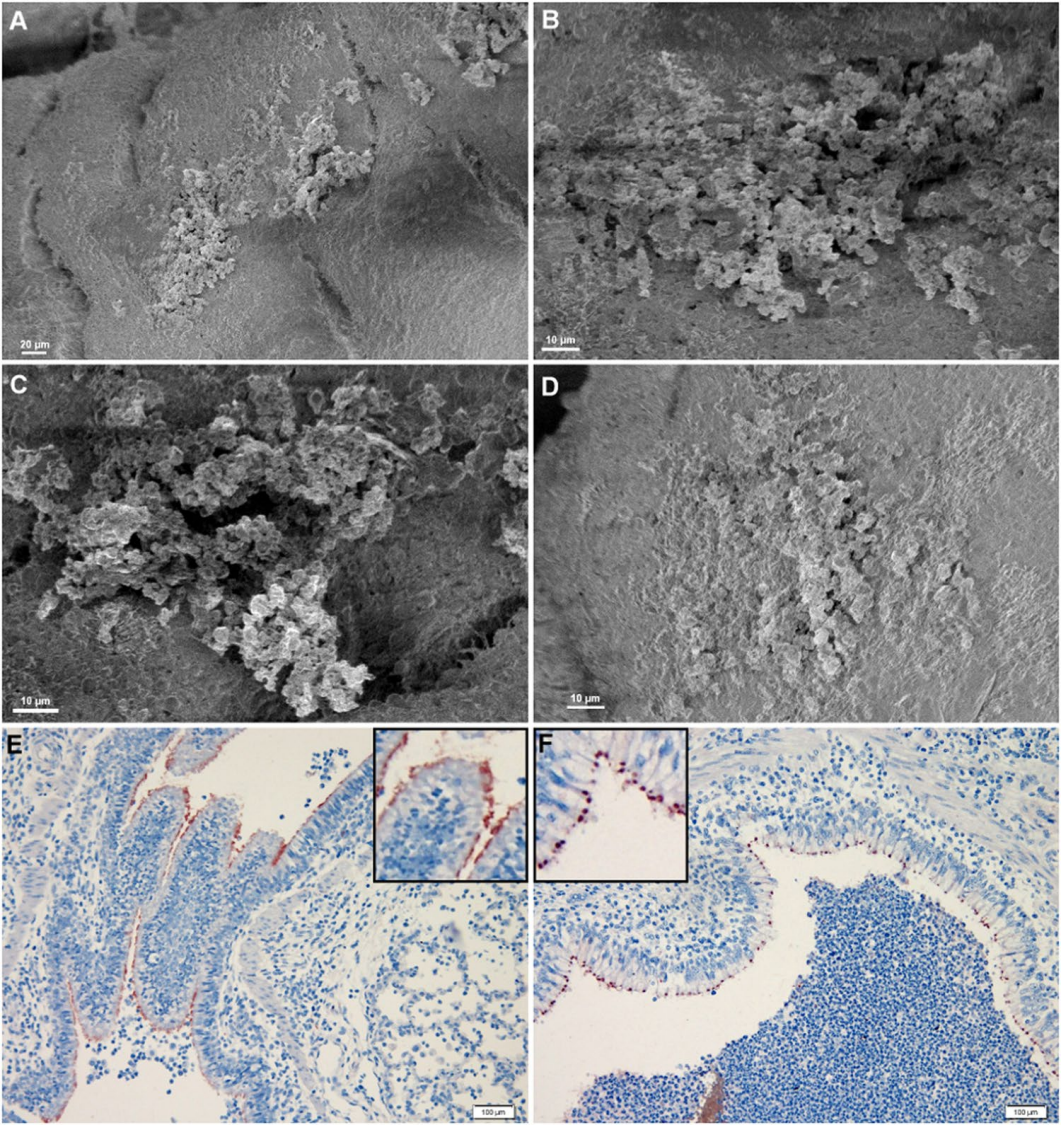
Biofilms of *M*. *hyopneumoniae* in swine respiratory tract. Panels A to D depict scanning electron micrographs of tracheal epithelium from pigs infected with the Hillcrest strain of *M*. *hyopneumoniae*. Biofilms, approximately 100 µm in diameter, were identified on the surface of epithelium denuded of cilia. All samples were imaged at 1 kV. Magnifications: (**A**) 600×; (**B**,**C**) 2000×; (**D**) 2500×. (**E**,**F**) IHC of bronchiolar epithelium from pigs infected experimentally (panel E) and naturally within a commercial piggery (panel F) with *M*. *hyopneumoniae*. *M*. *hyopneumoniae* cells adhere uniformly along the ciliary border (panel E), but in panel F the staining pattern is punctate and nodule like. This can be seen with more clarity in the inserts.

*M. hyopneumoniae* induces a chronic infectious state in the respiratory tract of swine, and pigs can be convalescent carriers of *M*. *hyopneumoniae* for up to 240 days post-challenge and remain infectious during this period^34^. In order to investigate whether or not *M*. *hyopneumoniae* can form biofilms within the respiratory tract, we examined tracheal sections of pigs experimentally infected with the *M*. *hyopneumoniae* strain Hillcrest, an Australian field isolate that is known to be highly infectious^35^. Strain J was not used as it is a lab-adapted strain that is not considered to be pathogenic. Using SEM, we detected large biofilms, approximately 100–150 µm in diameter on the surface of the respiratory tract epithelium of pigs euthanised 6 weeks post-infection (Fig. 1A–D). The epithelium was completely denuded of cilia; a hallmark of *M*. *hyopneumoniae* infection (Fig. 1A–D)^16^. Immunohistochemistry of sections of bronchiolar epithelium from the same batch of swine, infected experimentally with *M*. *hyopneumoniae* showed the pathogen localising at the ciliary border as expected in both acute (6 weeks after experimental infection; Fig. 1E) and in chronic infections in pigs from commercial swine production facilities (presumably infected for 3–4 months; Fig. 1F). Notably though, we observed that the staining pattern of *M*. *hyopneumoniae* cells differed between acute and chronically infected pigs; the latter exhibiting aggregates of staining at the ciliary border that resemble biofilms (insert in Fig. 1F).

### *M*. *hyopneumoniae* forms biofilms on abiotic surfaces

**Figure 2:**
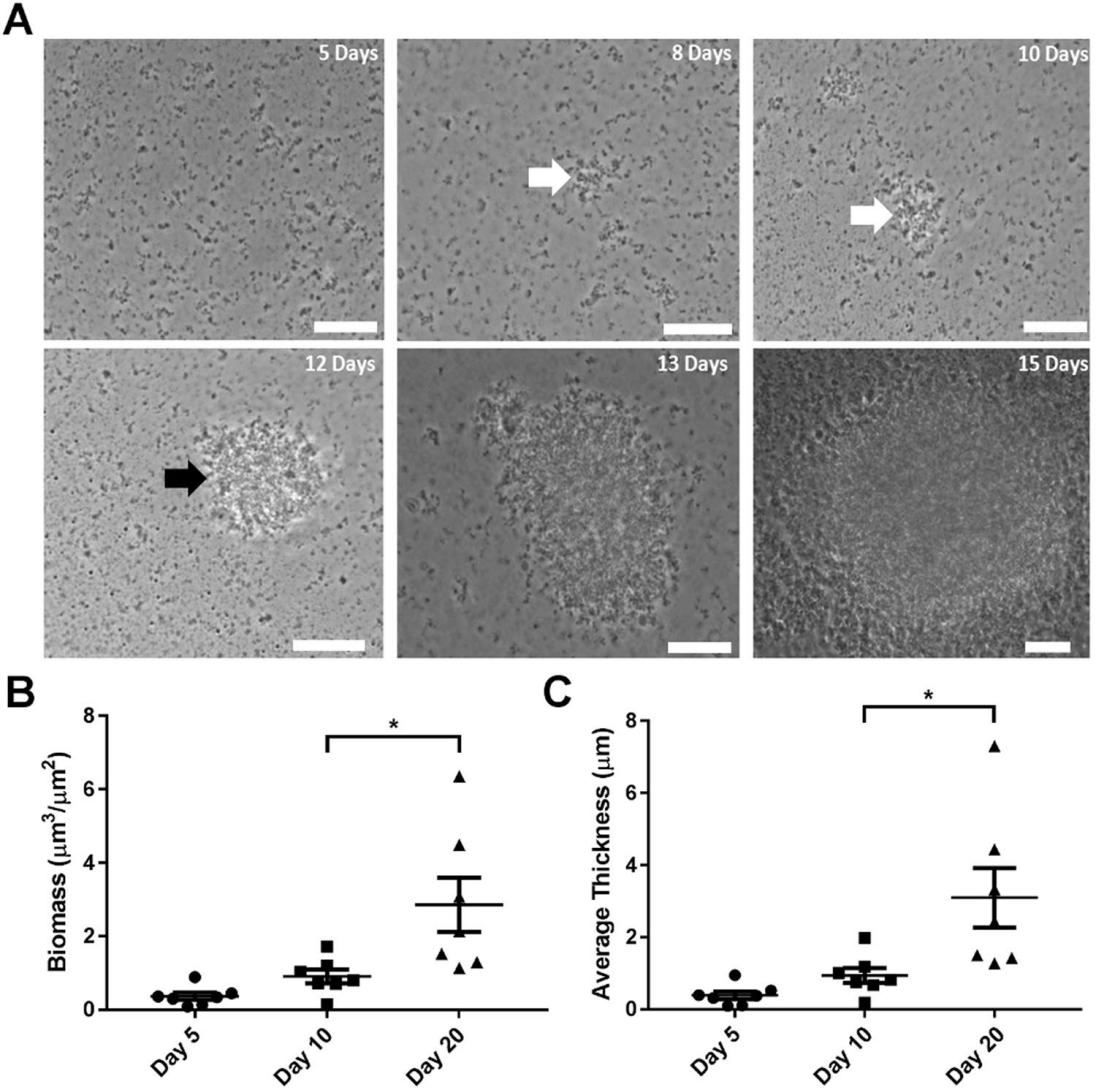
Biofilm formation of *M*. *hyopneumoniae* strain J cells on glass 5 to 20 days post inoculation (PI). (**A**) Within the first 5 days, only single *M*. *hyopneumoniae* cells can be seen adhering to the glass surface. Multilayered microcolonies were evident at 8 and 10 days PI (white arrows). Biofilms >20 µm in diameter were observed from day 12 (black arrows). Scale bar = 10 µm. (**B**,**C**) Increases in biomass and average thickness from days 5 and 10 to day 20 respectively were quantified using COMSTAT. Data represents the averages of 7 random fields of view of DAPI stained biofilms. *Indicates *P* < 0.05 generated by unpaired t-tests performed between Day 10 and Day 20.

*M*. *hyopneumoniae* has recently been shown to form biofilms on abiotic surfaces^26^ however, the mechanisms underpinning formation remains unknown. Prior to investigating these mechanisms, we sought to establish a reproducible protocol for promoting and investigating the formation of *M*. *hyopneumoniae* biofilms on abiotic surfaces. To promote biofilm formation, *M*. *hyopneumoniae* cells were grown in glass bottom FluoroDishes for 20 days with daily medium replenishment and monitored every 24 h using phase-contrast microscopy. Individual cells were observed to adhere to glass and interact with one another by day 5 (Fig. 2A). Discernible multilayered microcolonies <10 µm in diameter, estimated to be comprised of 8–15 *M*. *hyopneumoniae* cells, were observed 8–10 days post-inoculation (Fig. 2A). Not all cells appear to be in direct contact with one another suggesting that a matrix is formed around the cells of the growing microcolony. After 12 days, biofilms increased rapidly in size and were greater than 20 µm by day 13 (Fig. 2A), a significantly slower development of the biofilm compared to biotic surfaces.

COMSTAT^36^ was used to assess the biomass and average thickness, based upon the nucleic acid content of DAPI stained biofilms that formed between days 5, 10, and 20 (Supplementary Fig. S2) and imaged with confocal laser scanning microscopy (CLSM). Our data demonstrates that biofilms increase in both thickness and biomass significantly from day 10 to day 20; reflecting a shift from single adhering cells (days 5–10) to biofilms (>day 12) as indicated by our microscopy images (Fig. 2B,C).

### *M. hyopneumoniae* biofilms contain extracellular DNA

**Figure 3:**
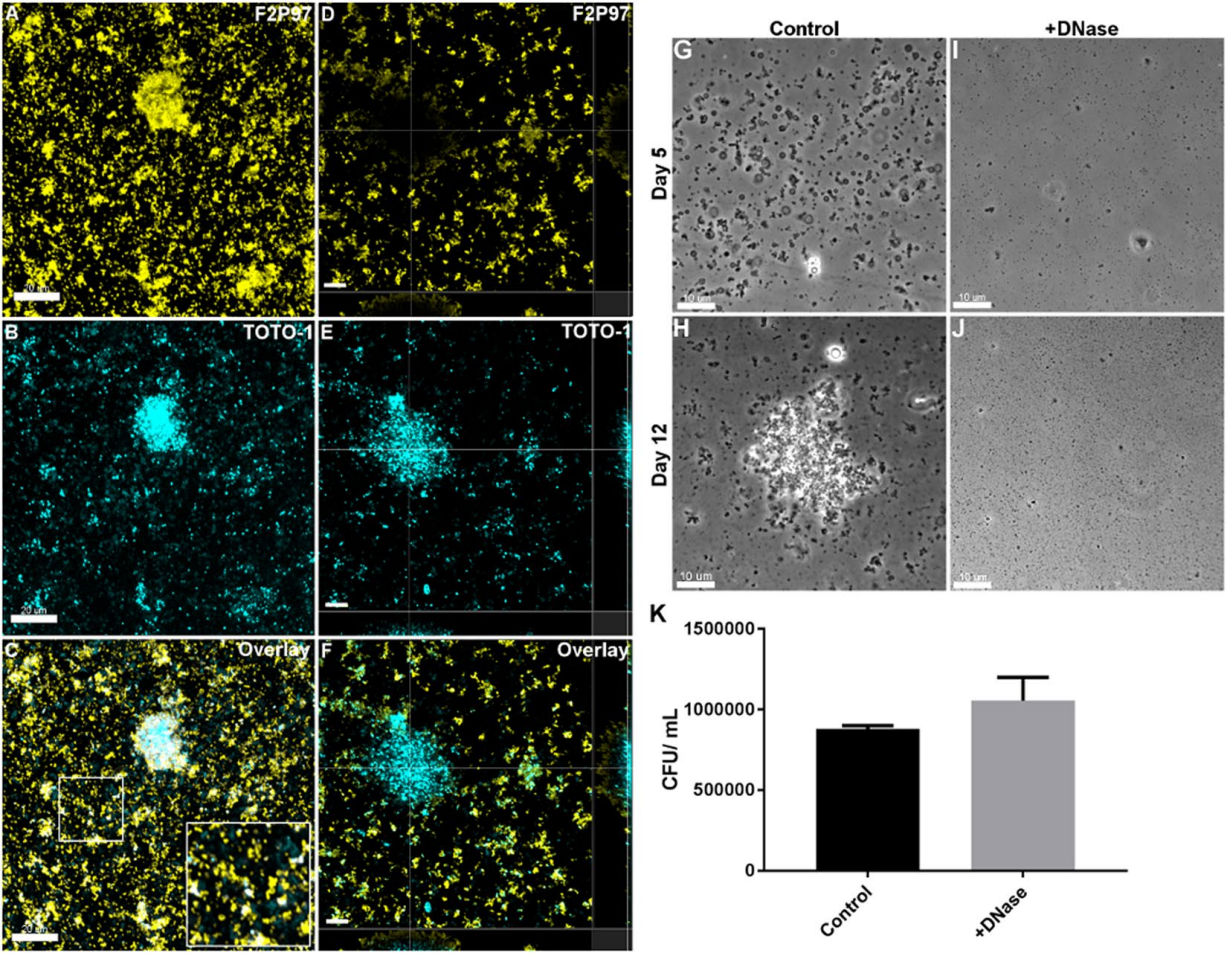
eDNA comprises part of the extracellular matrix and is crucial to the establishment of biofilms. Panels A and D show *M*. *hyopneumoniae* strain J cells 30 days PI labelled with rabbit F2_P94-J_ antisera and anti-rabbit CF 568 (labelled F2P97, yellow). Cells adhere to the glass surface and form biofilms. Panels B and E show eDNA stained with TOTO-1 (cyan). Panel C depicts an overlay of panels A and B. Panel C shows a *M*. *hyopneumoniae* biofilm that stains strongly with TOTO-1 and with rabbit F2_P94-J_ antisera. eDNA can also be seen in between the adhering *M*. *hyopneumoniae* cells and microcolonies (enlarged image insert). Panel D shows that some biofilms stain poorly with rabbit F2_P94-J_ antisera. Panels D–F show a section within a large, mature *M*. *hyopneumoniae* biofilm showing eDNA at the base of structure. *M*. *hyopneumoniae* cell that label with rabbit F2_P94-J_ antisera, albeit weakly, are positioned above the eDNA matrix. Panels G to J depict the effect of the nuclease Benzonase treatment (250 U/mL) on the establishment of biofilms on glass. Small microcolonies and individual cells attach to glass at day 5 PI (Panel G) and rapidly expand by 12 days PI (panel H). Panels I and J depict identical cultures of *M*. *hyopneumoniae* on glass in the presence of the nuclease. Biofilms were not observed when the culture medium was supplemented with nuclease. These images are representative of the total surface of the glass. (**K**) The effect of nuclease on the planktonic growth of *M*. *hyopneumoniae* cells. Cells were quantified as colony forming units after 7 days incubation on Friis agar. Nuclease treatment did not alter the growth characteristics of *M*. *hyopneumoniae* cells (*P* > 0.05). Scale bars in panels A–F and G–J are 20 µm and 10 µm respectively.

**Figure 4:**
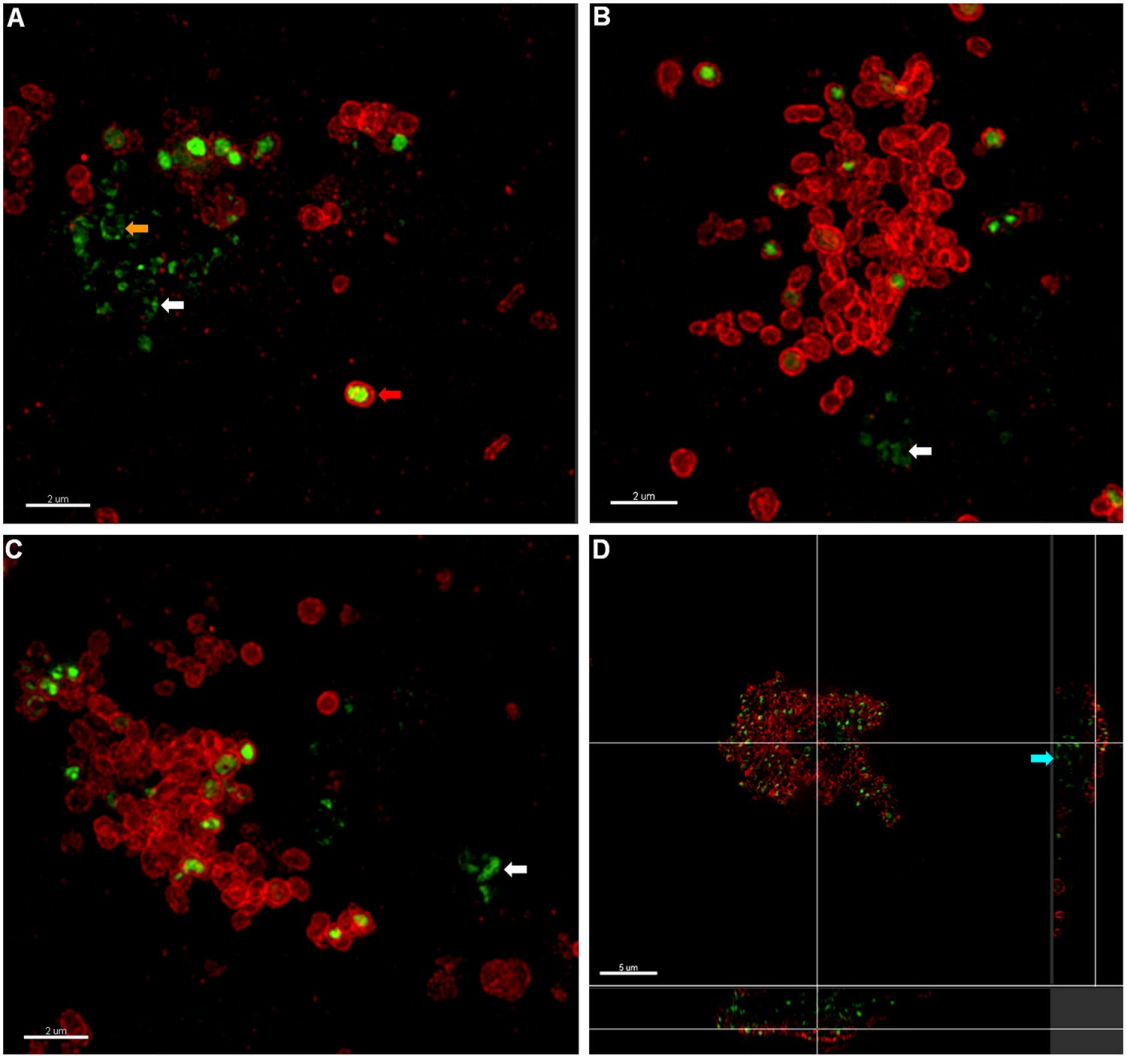
OMX 3D-SIM images of *M*. *hyopneumoniae* strain J biofilms on glass 30 days PI. *M*. *hyopneumoniae* cells were labelled with rabbit F2_P97_ antisera conjugated to anti-rabbit CF 568 (red) and eDNA was stained with TOTO-1 (green). Panels A to C show maximum intensity images of membranes of the *M*. *hyopneumoniae* cells residing within biofilms as well as the eDNA that has been released and deposited onto the glass (white arrows). Diffuse red fluorescence depicts to membrane remnants labelled with F2_P97_ antiserum. TOTO-1 accumulates inside a subpopulation of *M*. *hyopneumoniae* cells which appear to be in the process of degrading (red arrow). A circular staining pattern of eDNA, possibly from a recently lysed cell, can also be seen (orange arrow). (**D**) Orthogonal view of a *M*. *hyopneumoniae* biofilm demonstrating that the base of the biofilm is composed of eDNA/lytic cells (blue arrow). The cells on the top of the biofilm comprise intact *M*. *hyopneumoniae* cell membranes labelled with F2_P97_. Scale bars are 2 µm (panels A–C) and 5 µm (panel D).

Extracellular DNA is an important structural component of biofilms of many bacterial species^13,14^. To investigate if eDNA is present in *M*. *hyopneumoniae* biofilms, we used TOTO-1, a cell-impermeable nucleic acid stain and F2_P94-J_ antiserum to stain a 30-day old biofilm that formed on glass. We observed punctate staining with TOTO-1, both within biofilms and in microcolonies, suggesting that a proportion of *M*. *hyopneumoniae* cells that were bound to the glass have either lysed or have permeable membranes. Closer inspection of the surrounding area around the biofilms revealed that there was abundant eDNA in the spaces between adhering *M*. *hyopneumoniae* cells (Fig. 3A–C). eDNA appeared to form a base layer on the surface of the glass immediately beneath *M*. *hyopneumoniae* biofilms, microcolonies, and individual cells (Fig. 3D–F). This was most evident in mature biofilms where eDNA was seen in high abundance beneath the biofilm. 3D-SIM analyses showed eDNA in the area of the glass adjacent to biofilms as well as at the base of mature biofilms, consistent with confocal images depicted in Fig. 3F. The eDNA appears to be a product of cell decay as several cells simultaneously stained with TOTO-1 and F2_P94-J_ antisera (Fig. 4A–C). These data suggest that the membranes of subpopulations of *M*. *hyopneumoniae* cells become compromised and are in the process of cell decay. We propose that eDNA forms part of the conditioning material on the surface of the glass and serves as the foundation for biofilm formation.

In order to better understand the cellular composition of these biofilms we sought to examine the distribution of live and dead cells within 30-day old biofilms formed by *M*. *hyopneumoniae*. For this we used the cell-permeant nucleic acid dye SYTO 9 in combination with the cell-impermeant nucleic acid dye Ethidium Homodimer-1 (EthD-1). When overlayed, live and dead cells could clearly be differentiated from one another (Supplementary Fig. S3). While quantification wasn’t possible due to photolysis, the majority of cells within biofilms appeared viable, while dead cells could also be observed both within biofilms and in the surrounding area, although dead cells were sparsely distributed (Supplementary Fig. S3). EthD-1 also appeared to stain eDNA that had been deposited onto the glass slide, similarly to what was observed using TOTO-1 (Supplementary Fig. S3).

### Nuclease treatment inhibits biofilm formation

To determine the role, if any, of eDNA in the establishment of biofilms, we seeded *M*. *hyopneumoniae* cells into FluoroDishes in the presence (250 U/mL) or absence of the potent nuclease Benzonase. The medium supporting the growth of *M*. *hyopneumoniae* cells was replenished daily and Benzonase was maintained at 250 U/mL. After 5 days, control (no nuclease) cultures exhibited a large number of adherent cells (Fig. 3G), while cultures grown in the presence of the nuclease exhibited few or no adherent *M*. *hyopneumoniae* cells (Fig. 3I). These data suggest that eDNA is required for individual cells and small microcolonies to adhere to glass. After 12 days post-inoculation, rapidly growing biofilms were evident in control cultures (Fig. 3H), while cultures grown in the presence of nuclease exhibited no evidence of biofilm formation (Fig. 3J). To determine if nuclease adversely affects growth of *M*. *hyopneumoniae*, planktonic broth cultures were exposed to nuclease for 24 h and colony forming units (CFU) were determined by plating onto Friis-agar. We were unable to detect an inhibitory effect on *M*. *hyopneumoniae* cells that were cultured in the presence of nuclease compared with control cultures (no nuclease). A slightly elevated number of viable cells that were treated with nuclease was observed, however the effect was not statistically significant (Fig. 3K).

### Pleomorphic cell types among subpopulations of *M. hyopneumoniae* cells

**Figure 5:**
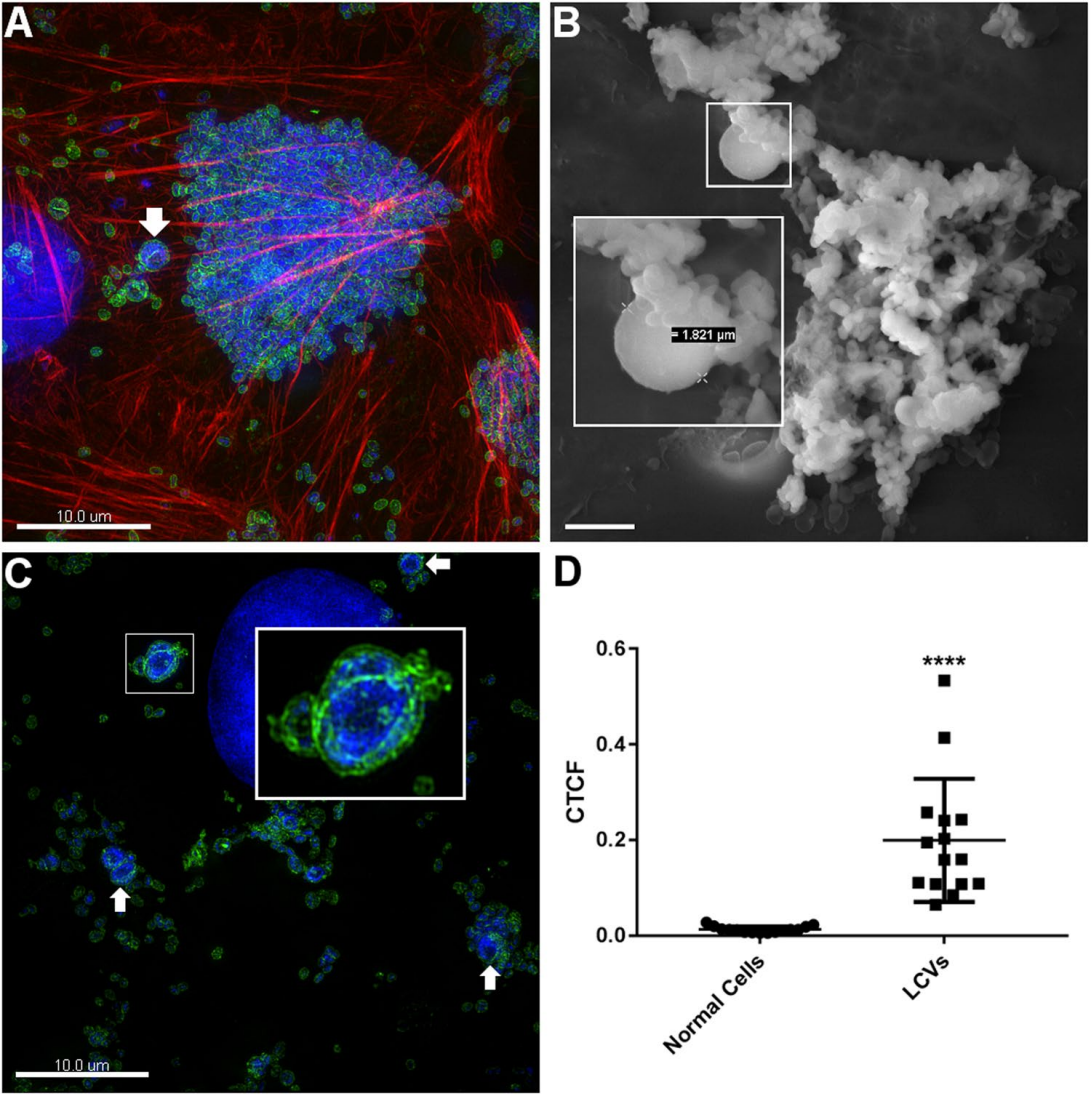
Large cell variants of *M*. *hyopneumoniae* strain J. Panels A and C depict OMX 3D-SIM images of *M*. *hyopneumoniae* (labelled with F2_P97_ antisera conjugated to anti-rabbit CF 488 (green)) adhering to PK-15 monolayers. Note the LCV identified by the white arrow in panel A (scale bar = 10 µm.). The cytoskeleton of PK-15 cells is stained with Phalloidin-CF 568. Panel B shows an SEM image of an *M*. *hyopneumoniae* biofilm with a LCV ~1.8 µm in diameter (white box) (scale bar = 2 µm.). LCVs depicted in panels A and C (white arrows and boxed areas) stain intensely with DAPI. Panel C demonstrates an LCV that may have membrane blebs/vesicles attached to it. LCVs accumulate more DAPI stain compared to conventional *M*. *hyopneumoniae* cells (panel D). The image depicts a scatter plot of corrected total cell fluorescence (CTCF), with the standard error of the mean, of normal cells and LCVs. ****Indicates *P* < 0.0001 generated from an unpaired t-test.

*M*. *hyopneumoniae* cells normally adopt a fairly uniform cell size ranging from 400–1200 nm in diameter, but typically average 500 nm^22^. However, our confocal and live-cell experiments suggest that Large Cell Variants (LCVs) exist in the *M*. *hyopneumoniae* population with diameters upwards of 2 µm. In confocal images of *M*. *hyopneumoniae* cells adhering to PK-15 cells, we observed numerous examples of LCVs (Fig. 5A). LCVs were also observed by SEM within a biofilm forming on the surface of PK-15 cells (Fig. 5B) and were also strikingly evident in 3D-SIM images of *M*. *hyopneumoniae* in contact with PK-15 cells (Fig. 5C). A septum-like structure that stained with F2_P97_ antisera stretches along the length of LCVs (Fig. 5C). LCVs stain intensely with DAPI (Fig. 5A,C) suggesting that they contain elevated amounts of DNA compared with smaller, typical cell types in the population. To quantify the difference in DNA content between LCVs and regular-sized cells, we compared the relative level of fluorescence in a given cell, also known as corrected total cell fluorescence, of *M*. *hyopneumoniae* cells stained with DAPI, using 3D-SIM. Data from 30 cells, across three fields of view, showed LCVs contained 15 times more DNA (stained with DAPI) than typical *M*. *hyopneumoniae* cells (Fig. 5D). Notably, when *M*. *hyopneumoniae* was cultured on abiotic surfaces, LCVs were observed with less frequency after biofilms were established, suggesting that they play a role in the early stages of biofilm formation. Smaller morphological variants were often seen in association with LCVs (Fig. 5C) and these were also observed to bud from LCVs in real time (described later).

**Figure 6:**
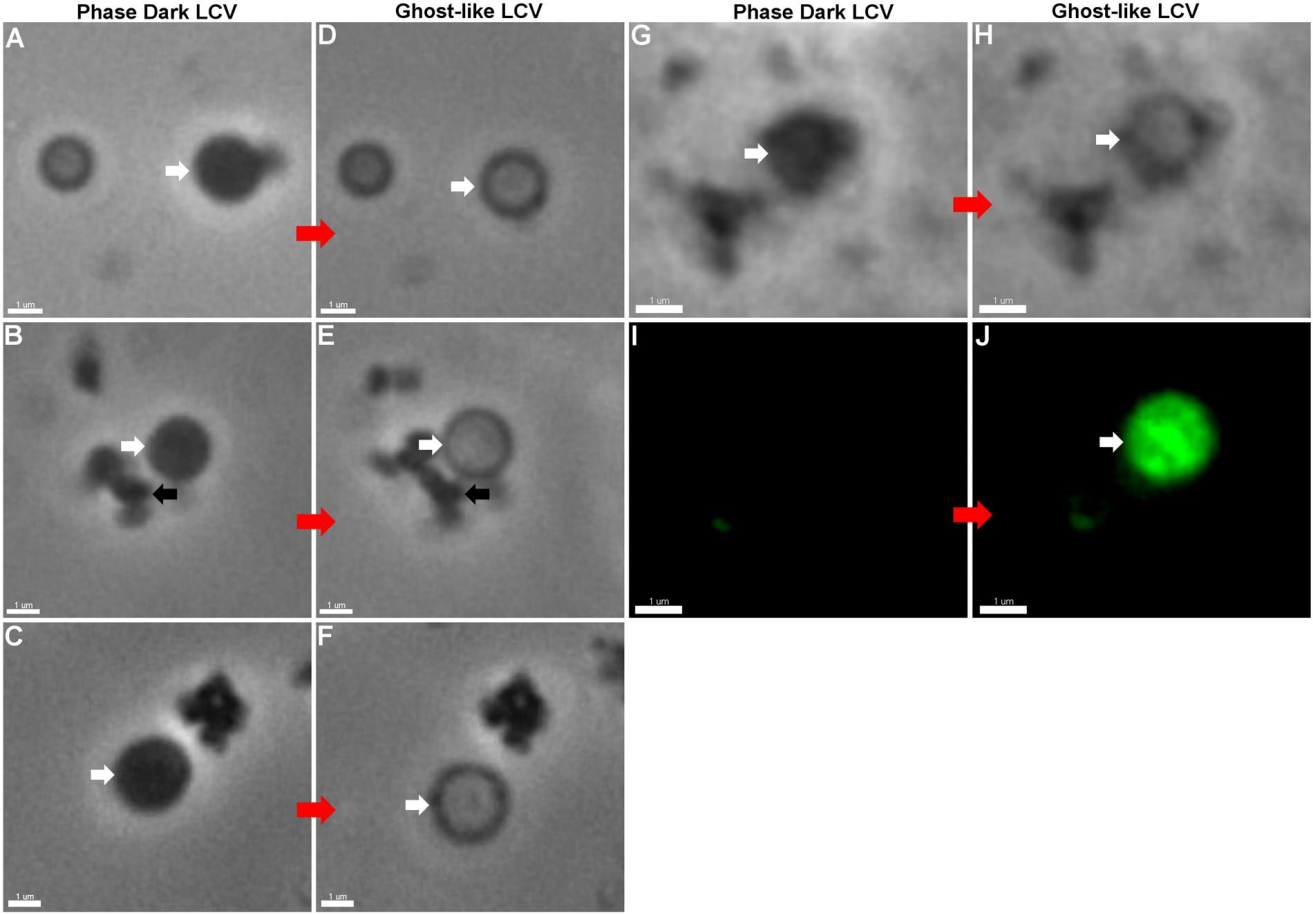
Time-lapse microscopy depicting LCVs transitioning to ghost cells. Panels A–C show phase contrast images of three LCVs adhering to WPI dishes. Panels D-F show LCVs in panels A–C undergoing morphological changes becoming translucent cell types within a short time period (5 minutes). Panel G depicts a phase dark cell, that does not stain with TOTO-1 (panel I), which transitions to a translucent cell type (panel H) and readily accumulates TOTO-1 (panel J, white arrow). Smaller morphological variants can be observed to bud from the LCV during the transition (black arrows). White arrows throughout the figure indicate the corresponding phase dark, and ghost-like LCVs.

The behaviour of LCVs adhering to abiotic surfaces was investigated in real-time, using time-lapse microscopy. LCVs were observed to transition from their typical, phase-dark appearance, to translucent “ghost-like” cells (Fig. 6A–F), creating several smaller ‘daughter’ cells approximately 500 nm in diameter (Supplementary Video S1). These events, observed on numerous occasions, were spontaneous and unpredictable. As LCVs transformed to ghost cells they accumulated TOTO-1 (Fig. 6G–J), presumably due to their membranes becoming compromised. Breaches in the membranes of LCVs, in the process of transitioning to ghost cells, were identified in time-lapse images (Supplementary Fig. S4) and these may be sites from where eDNA is released. LCVs often collapsed when transitioning to ghost cells, releasing cellular content into the surrounding milieu (Supplementary Video S2). In one example, an LCV appears to undergo an explosive lysis event, creating what appears to be membrane vesicles (Supplementary Video S3). Prolonged fluorescence imaging of TOTO-1 stained *M*. *hyopneumoniae* cells appeared to induce photolysis and therefore this was avoided. This experimental issue made it a challenge to capture events associated with the release of eDNA. *M*. *hyopneumoniae* cells less than 1 µm in size were never observed to undergo similar lysis or transition events. LCVs did, however, generate small (<0.5 µm) cellular morphotypes that rapidly bud from the membrane surface (Fig. 6E). These budding events were captured in movies depicting LCV transitioning to ghost cells after undergoing cell division (Supplementary Video S1). We do not know if these morphotypes are capable of independent cell replication.

## Discussion

Here we provide the first ever *in vivo* evidence of biofilm formation in the porcine pathogen *M*. *hyopneumoniae* and outline a time course of events depicting *M*. *hyopneumoniae* forming biofilms on an abiotic surface. Biofilms form on glass only after prolonged incubation (10–12 days) but form rapidly on cell monolayers suggesting that *M*. *hyopneumoniae* is unable to adhere to glass without a conditioning phase. eDNA appears to be essential for initiating biofilm formation during this conditioning phase. The eDNA assembles at the base of microcolonies and biofilms (Fig. 3F) presumably to provide a matrix for *M*. *hyopneumoniae* adherence. 3-D SIM images (Fig. 4) of microcolonies and biofilms identify a subpopulation of *M*. *hyopneumoniae* cells in these structures that accumulate the cell-impermeable dye, TOTO-1, indicative of these cells having a compromised membrane structure. We show *M*. *hyopneumoniae* cells that stain with TOTO-1 show membrane irregularities and eDNA leaking out into the surrounding environment (Fig. 4B,C). The nuclease Benzonase was effective in inhibiting biofilm formation on glass surfaces but did not influence the growth of *M*. *hyopneumoniae* cells (Fig. 3K). eDNA is a known important component of the extrapolymeric matrix of many microbial biofilms^37–39^ and collectively, these data indicate that the conditioning film required for the formation of a biofilm on glass is comprised of eDNA. Although eDNA has never been directly demonstrated to play a role in mycoplasma biofilm formation, the extrapolymeric matrix of *Mycoplasma pulmonis* biofilms have been shown to contain protein, lipid, DNA, and polysaccharides^6^. While our data is consistent with observations of eDNA release in walled bacterial pathogens^40^, we show for the first time that Large Cell Variants (LCVs) of *M*. *hyopneumoniae* may be a source of eDNA that initiates the process of biofilm formation in Mollicute species.

Based upon fluorescence intensity, LCVs appear to contain a significantly higher nucleic acid content than typical *M*. *hyopneumoniae* cell morphotypes (Fig. 5C,D). LCVs appear to release eDNA during the process of transitioning to ghost cells. The transition event captured using time-lapse microscopy is rapid (within minutes) and ghost cells readily accumulate TOTO-1, indicating that their cell membranes become compromised during the transition process. Time-lapse images depicting breaches in ghost cell membranes that appear during the transition phase, provide additionaly data to support this hypothesis (Supplementary Fig. S4). Ghost cells are transient, often degrade rapidly (Supplementary Video S2) and occasionally undergo explosive, lytic events (Supplementary Video S3). LCVs appear less frequently after biofilms become established at about 12 days post-inoculation, but further studies are needed to quantify this, as it could be a limitation of our experimental approach that masks their detection. These observations suggest that the selective pressure or signalling events that regulate LCV formation diminish once a mature biofilm forms. Due to phototoxicity associated with imaging fragile wall-less bacteria such as *M*. *hyopneumoniae*, in conjunction with the prolonged period taken to culture LCVs, and the time (hours) taken for LCVs to undergo lysis, it was difficult to capture the event that depicts the release of eDNA using time-lapse microscopy. Notably, LCVs also adhere to PK-15 monolayers and stain with F2_P94-J_ antisera indicating that they display adhesins that recognise epithelial cell receptors however, further studies will be needed to investigate the role of eDNA release by LCVs in biotic biofilms. F2_P94-J_ antisera labelled a septum-like structure in LCVs (Fig. 5A,C), indicating that the cell surface architecture is remodelled in these morphotypes, a process that results in the redistribution of surface proteins. Morphotypes in other *mycoplasma* spp., such as the human pathogen *Mycoplasma pneumoniae*, have been noted. These morphotypes, termed spherules, have been depicted occurring within *M*. *pneumoniae* biofilms^41,42^. It has also been noted that supplementing the growth media of *Mycoplasma spp* with long chain fatty acids can induce morphological variants^43^, further supporting the notion that membrane remodelling plays a role in the formation of LCVs in *M*. *hyopneumoniae*. It appears that the presence of distinct morphotypes in mycoplasmas, much like L-forms for walled-bacteria, represent a potentially important and understudied life stage.

Host cell surfaces provide a nutrient environment that is conducive to the proliferation of metabolically challenged, genome-reduced pathogens. *M*. *hyopneumoniae* cannot synthesise amino acids, nucleic acids, lipids and has only a limited capacity to generate ATP from glycolysis^44^. *M*. *hyopneumoniae* devotes almost 5% of its limited coding capacity to P97 and P102 gene families and their expression is likely to play an important role in biofilm formation on epithelial cells within 16 h of inoculation. Processing events in these paralog families generate an impressive arsenal of adhesive proteoforms that are adept at binding to a variety of host molecules^29–32,45–54^. These adhesins display short linear motifs enriched in positively charged amino acids, such as those that bind heparin^28–32,45,46,48–50,52,54–56^. These motifs promote binding to anionic molecules, including glycosaminoglycans, that decorate the surfaces of epithelial cell proteoglycans. Proteins that bind heparin often share features with those that bind DNA and are likely to facilitate cell-cell and cell-matrix adhesion.

Our *in vitro* and *in vivo* studies suggest that *M*. *hyopneumoniae* persists in the lungs of pigs and assists in initiating a chronic infectious state by forming biofilms in the swine respiratory tract. The formation of these biofilms, both by *M*. *hyopneumoniae* and other species of mycoplasma, provides a mechanism to resist the lytic effects of antimicrobials and the host immune response^5,8,26^. In addition to forming biofilms in its host, *M*. *hyopneumoniae* has been shown to be encapsulated within microaerophilic mucosal droplets, capable of long distance transmission on air currents^1,2^. Furthermore, a recent study demonstrated that *M*. *hyopneumoniae* cells can survive for up to 8 days at 4 °C on abiotic surfaces^27^; conditions that mimic those found within intensive swine farms as well as those in the environment with natural populations of wild pigs. Our findings suggest that the wall-less pathogen, *M*. *hyopneumoniae* relies on the generation of eDNA to form biofilms on abiotic surfaces, a mechanism that may be essential for survival outside the host.

In conclusion, we show *M*. *hyopneumoniae* forming prolific biofilms on abiotic surfaces, host cell monolayers, and on the denuded epithelium of the swine respiratory tract. For the first time, we show that biofilms that form on abiotic surfaces require eDNA that is released from sub-populations of *M*. *hyopneumoniae* cells. Most notable among these subpopulations are LCVs that are up to four times larger than typical *M*. *hyopneumoniae* cells and contain up to 15 times more DNA. LCVs undergo changes to their morphology, generate small-cell variants of unknown function, decay rapidly, and are the most likely source of e-DNA (and cell contents). These findings have significant implications for understanding the biology of *M*. *hyopneumoniae*. Future studies will focus on the identification of biofilm-associated genes that may benefit vaccine development in order to alleviate the use of antimicrobials to control infections caused by *M*. *hyopneumoniae*.

## Materials and Methods

[All data generated or analysed during this study are included in this published article (and its Supplementary Information files)].

### Bacterial strains and culture

All *in vitro* experiments were performed using the lab adapted *M*. *hyopneumoniae* strain J, and the experimental infection of pigs was performed using the Hillcrest strain. Both *Mycoplasma hyopneumoniae* strains J and Hillcrest were grown in liquid Friis medium overnight at 37 °C as previously described^57^, with no modifications. Overnight cultures, at mid-exponential phase, contain approximately 5 × 10^5^–1 × 10^6^ CFU/mL. For the culturing of *M*. *hyopneumoniae* cells onto solid media, Friis agar was prepared as previously described^58^, with no modifications. For plating, overnight *M*. *hyopneumoniae* strain J cultures were washed twice in PBS by centrifugation at 11, 000 × g and resuspended in warm Friis medium. Cell suspensions were diluted in Friis broth, vortexed briefly to separate aggregates, and spotted onto the agar by pipetting. The spots were allowed to air dry before being incubated at 37 °C/5% CO_2_ for at least 7 days.

### Culturing of *M. hyopneumoniae* biofilms

Biofilm growth was encouraged by diluting a 48 h *M*. *hyopneumoniae* culture 1:100 in Friis media. Aliquots (1 mL) of the diluted culture were inoculated into 35 mm FluoroDishes (World Precision Instruments) and incubated at 37 °C. After an initial 48 h the spent Friis medium was replaced with new media and this process was repeated every 24 h for a minimum of 12 days or until mature biofilms could be readily observed. This process was also performed in parallel with uninoculated Friis media as a negative control.

### Treatment of *M. hyopneumoniae* cultures with nuclease

Biofilm formation was promoted in FluoroDishes in an identical manner as described above, with the exception that the Friis media was supplemented with 250 U/mL of Benzonase (Sigma-Aldrich) that had been filter sterilised through a 0.22 µm filter. Experiments were performed in triplicate in addition to control FluoroDishes that received Friis media without nuclease (to simulate normal biofilm growth).

To investigate the effect of nuclease on the planktonic growth of *M*. *hyopneumoniae* strain J, a single overnight culture was subcultured into two identical cultures, in the presence or absence of 250 U/mL of the nuclease Benzonase (Sigma-Aldrich). After overnight growth, each culture was diluted to 10^−1^ and 10^−2^ in fresh Friis broth and inoculated onto Friis agar as described above. CFU/mL were presented as the average of duplicate spots, with the standard error of the mean and unpaired t-test calculated using GraphPad Prism.

### Live-cell imaging

Dishes were monitored daily using a Nikon Ti-E inverted microscope. This phase contrast system was used to image the dishes under 100× oil immersed magnification. Images were captured using a Photometrics Cascade: 1 K EMCCD Imaging Camera (Coherent Scientific). These images were further processed using NIS Elements freeware (Nikon Instruments).

### Adherence of *M. hyopneumoniae* cells to PK-15 monolayers

Experiments were performed as described previously^51^.

### Immunofluorescence microscopy of infected monolayers

Experiments were performed as described previously^51^. Samples were imaged on a Nikon A1 Confocal Laser Scanning Microscope. These samples were also imaged using super-resolution 3D-SIM on a V3 DeltaVision OMX 3D-SIM Imaging System (Applied Precision, GE Healthcare) as previously described^59^.

### Immunofluorescence microscopy of biofilms

All immunofluorescence steps conducted below were followed by washing 3× with sterile PBS. All dilutions were made in 1% BSA/PBS unless otherwise stated. Once mature biofilms were observed, the culture dish was washed 3× with sterile PBS and fixed with ice cold 100% methanol for 30 s. Non-specific binding sites were blocked with 2% BSA in PBS for 1 h. Polyclonal rabbit antisera raised against the major surface and immunogenic P65 lipoprotein diluted 1:100 was incubated for 1 h. A 1:1000 dilution of Alexa Fluor 568 (Invitrogen, Life Technologies) was incubated for 1 h. DAPI (Life Technologies) was incubated at 10 μM for 30 min. Samples were imaged on a Nikon A1 Confocal Laser Scanning Microscope.

For the staining of eDNA, *M*. *hyopneumoniae* strain J cells were cultured in 35 mm FluoroDishes as described above. For eDNA staining, biofilms were washed 3× with sterile PBS, followed by staining with the membrane-impermeable nucleic acid dyes TOTO-1 Iodide (Life Technologies) at a final concentration of 1 µm for 1 h at RT. Excess dye was removed by washing 3× with sterile PBS, followed by fixation in 2% paraformaldehyde for 1 h at RT. Excess aldehydes were quenched in 100 mM glycine for 5 min at RT, followed by blocking in 2% BSA from 1 h at RT. *M*. *hyopneumoniae* cells were labelled using polyclonal rabbit antisera raised against the R1 and R2 regions of P94_J_ (F2_P94-J_)^48^ at a dilution of 1:100 for 1 h at RT. This was followed by adding a 1:1000 dilution of CF goat anti-rabbit 568 for 1 h at RT. Following extensive washing, the PBS was removed and replaced with VECTASHIELD. For live/dead staining of biofilms, a duplicate dish of the aforementioned FluorDish was washed 3× with sterile PBS, followed by staining with the membrane-permeant nucleic acid dye SYTO 9, and the membrane-impermeant nucleic acid dye Ethidium Homodimer-1 (EthD-1) at a final concentration of 5 µM and 1 µM respectively, for 1 h at RT. Following washing in PBS, the FluoroDish was immediately imaged on a Nikon A1 Confocal Laser Scanning Microscope.

### Processing of immunofluorescence images

Images captured with the Nikon A1 Confocal Laser Scanning Microscope (CLSM) and those generated by the DeltaVision OMX 3D-SIM were processed using Bitplane, Imaris Scientific 3D/4D image processing software to create Maximum Intensity Projection (MIP) and slices images.

For quantitative analysis, 5 and 20 day biofilms were fixed, stained with DAPI at a final concentration of 10 µM and imaged using CLSM using the 40× objective. Raw image Z-stacks were converted to TIF series using Imaris software (Bitplane Scientific Solutions, Zurich, Switzerland). These files were analysed by COMSTAT, written as a script by Arne Heydorn (Center for Biomedical Microbiology, BioCentrum, Technical University of Denmark) in MATLAB (The MathWorks Inc., Natick, Massachusetts). The parameters measured were total biomass and average thickness. Data is presented as the average of seven random fields of view, with the standard error of the mean and unpaired t-test calculated using GraphPad Prism.

For the quantification of the difference in nucleic acid content between typical *M*. *hyopneumoniae* cells and LCVs, fifteen DAPI (final concentration of 10 µM) stained (paraformaldehyde-fixed samples) *M*. *hyopneumoniae* and LCVs (30 total) across three fields of view, were imaged using 3D-SIM. The area, integrated density, and mean grey value of each cell was then calculated using ImageJ software^60^. The Corrected Total Cell Fluorescence^61^ was then calculated by subtracting the multiple of the area of the selected cell and mean fluorescence of the background, from the integrated density. The data is presented as a scatter plot, with the stand error of the mean and unpaired t-test calculated using GraphPad Prism.

### Processing for SEM

Preparation of infected monolayers for SEM was performed as previously described^51^, with no modifications.

### Immunohistochemistry

All animal procedures were approved by the Animal Ethics Committee at the Elizabeth Macarthur Agricultural Institute and were in accordance with the *Australian Code of Practice for the Care and Use of Animals for Scientific Purposes*. For experimental infections, pigs were inoculated endotracheally with 10 mL of *M*. *hyopneumoniae* strain Hillcrest culture containing 1–2 × 10^13^ CCU/mL as previously described^35,52^. Pigs were monitored daily and euthanized 6 weeks post infection. Abattoir pigs, infected naturally with *M*. *hyopneumoniae* within a commercial piggery, were 24 weeks of age at slaughter and presumed to be infected for 3–4 months. *M*. *hyopneumoniae* infection was initially detected via qPCR as described previously^35,62^, and qPCR positive samples were used for immunohistochemistry. Serial sections of lung lesions from pigs experimentally infected with *M*. *hyopneumoniae*^35,52^, as well as lung tissue from abattoir pigs were examined for *M*. *hyopneumoniae* distribution using immunohistochemistry as previously described with no modifications^49,52^.

### SEM of tracheal sections

Tracheal sections from the same pigs that were used for immunohistochemistry were harvested and prepared for SEM as previously described^63^.

## Electronic supplementary material

Supplementary Data

Supplementary Video S1

Supplementary Video S2

Supplementary Video S3
