# Supplementary material for: Extracellular DNA release from the genome-reduced pathogen Mycoplasma hyopneumoniae is essential for biofilm formation on abiotic surfaces
Source: Sci Rep. 2018 Jul 10;8:10373. doi: 10.1038/s41598-018-28678-2 (PMC6039474; doi:10.1038/s41598-018-28678-2)
Supplement: Supplementary file 1 — Supplementary Data [file 41598_2018_28678_MOESM1_ESM.docx]

Supplementary Material

Extracellular DNA release from the genome-reduced pathogen *Mycoplasma hyopneumoniae* is essential for biofilm formation on abiotic surfaces

Benjamin B.A. Raymond, Cheryl Jenkins, Lynne Turnbull, Cynthia B. Whitchurch and Steven P. Djordjevic*

* Corresponding author: Steven. P. Djordjevic


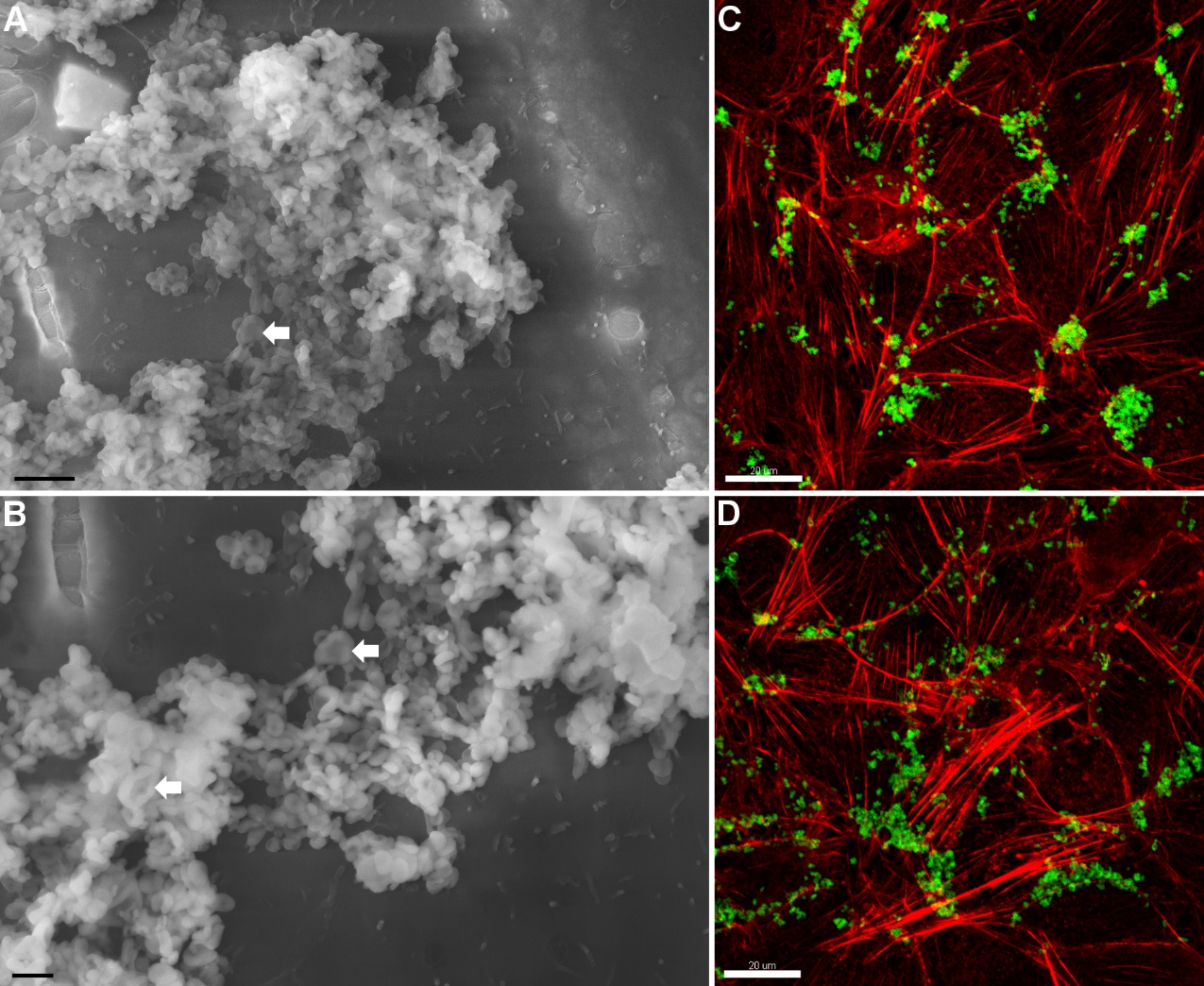


Supplementary Figure S1: Biofilm formation on PK-15 cells. *M. hyopneumoniae* cells adhering to PK-15 16 h PI followed by SEM and confocal microscopy. A-B) SEM images showing large aggregates of *M. hyopneumoniae* cells adhering to the surface of PK-15 monolayers. White arrows indicate pleomorphic *M. hyopneumoniae* cells within the biofilm. Scale bars are 2 µm and 1 µm in panels A and B respectively. C-D) Confocal micrographs demonstrating *M. hyopneumoniae* cells (labelled with F2_P94-J_ antisera) adhering to PK-15 monolayers. The pattern of colonisation on PK-15 cells is complex. *M. hyopneumoniae* cells form biofilms along strands of filamentous actin, in regions where actin nodes converge, and in regions devoid of Phalloidin. Phalloidin CF™ 568 was used to stain actin. Scale bars in panels C and D are 20 µm.


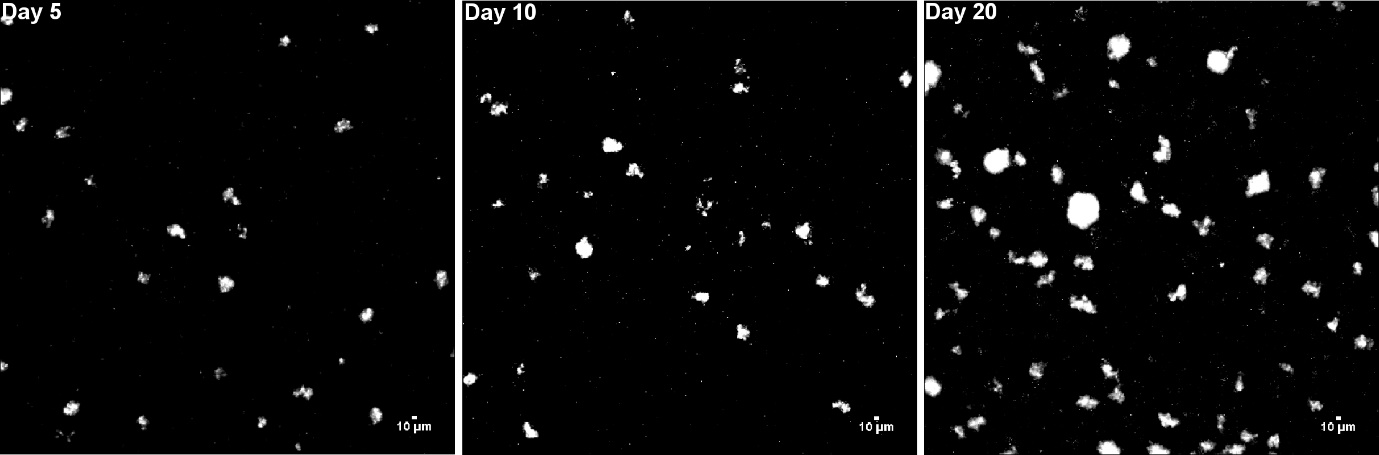


Supplementary Figure S2: Representative binary images of *M. hyopneumoniae* strain J cells adhering to glass surfaces at different time points and imaged using confocal microscopy. Days are indicated in the top left-hand corner. White areas correspond to DAPI-stained *M. hyopneumoniae* cells within microcolonies and/or biofilms.


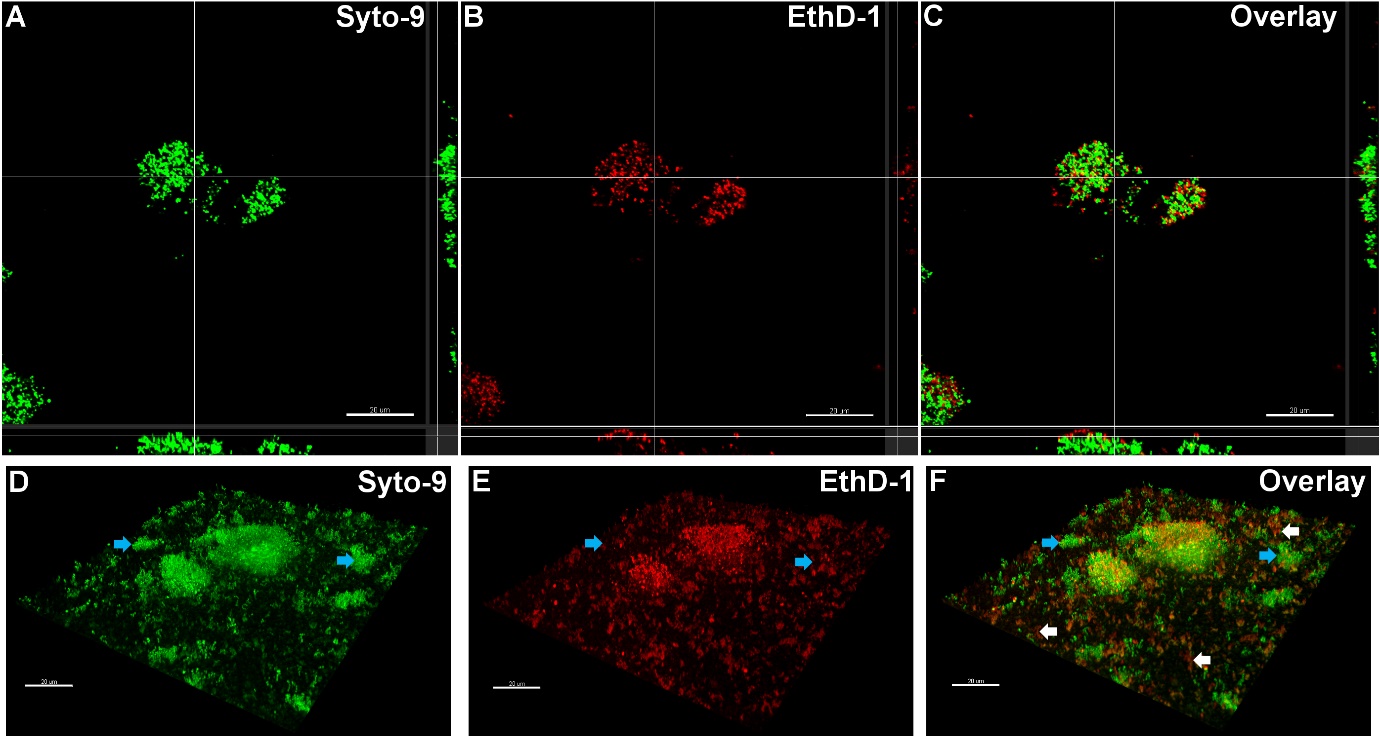


Supplementary Figure S3: Distribution of live/dead cells within mature *M. hyopneumoniae* biofilms. Thirty-day old biofilms were stained with Syto-9 and EthD-1 and imaged using confocal microscopy. In panels A and D, viable cells have been stained with the membrane-permeant nucleic acid dye SYTO 9 and appear green. In panels B and E, dead/dying cells have been stained with the membrane-impermeant dye EthD-1 and appear red. In panels C and F, the overlay of the respective panels shows the distribution of viable (green) and dead (yellow/red) *M. hyopneumoniae* cells within the biofilm. A-C) Orthogonal view demonstrating the distribution of viable and dead cells within a *M. hyopneumoniae* biofilm. The dead cells can be seen to reside within the spaces between clusters of viable cells. D-F) Volume view of a field of view demonstrating mature biofilms as well as a lawn of *M. hyopneumoniae* cells. A large biofilm can be seen in the center of the images that stains intensely with Syto-9 but also contains EthD-1-staining dead/dying cells. Small microcolonies do not seem to contain as many dead cells as the larger biofilms (blue arrows). The combined staining of SYTO 9 and EthD-1 appeared to also stain eDNA that had been deposited onto the coverslip (white arrows), similarly to what we observed with TOTO-1. Scale bars are 20 µM.


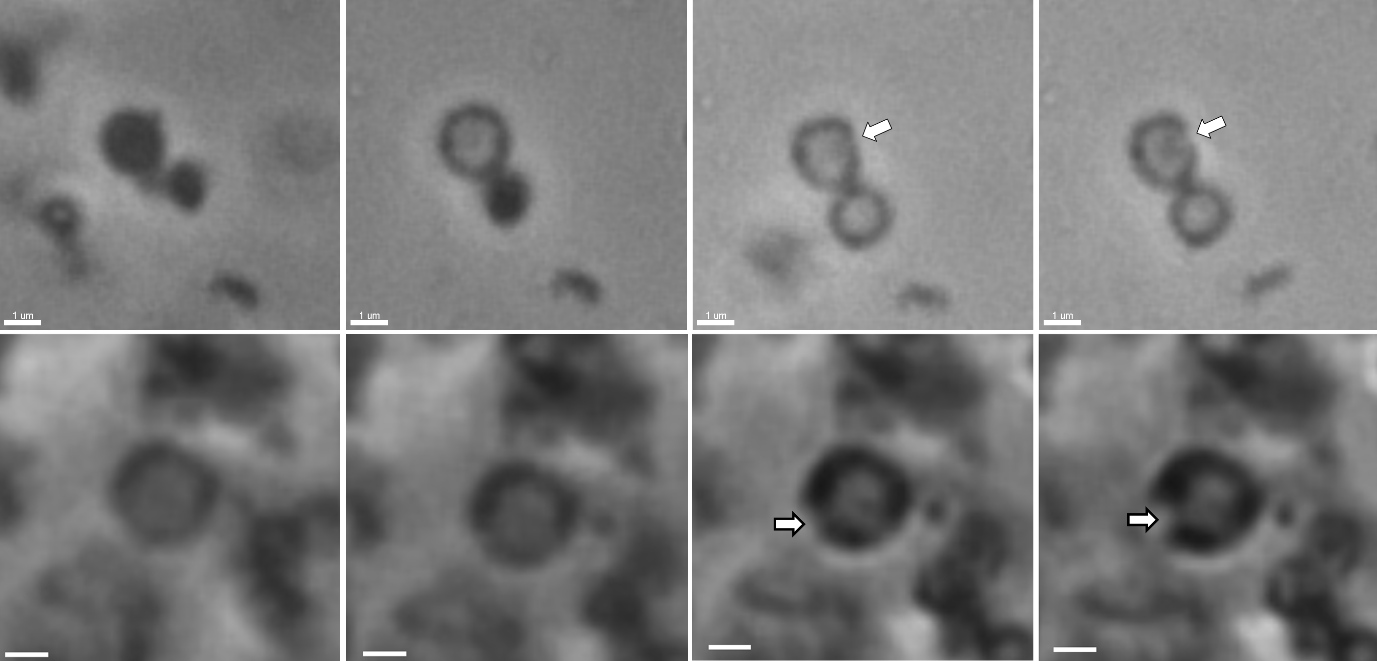


Supplementary Figure S4: Appearance of a breach in the membrane in two LCVs (top and bottom). In the first example, the LCVs can be seen converting to a ghost cell, followed by the formation of a membrane breach (white arrow) over the course of approximately 10 h. A similar observation can be seen in the second example (white arrow). Scale bar is 1 µm.

Supplementary Video S1: Cell division in LCVs. A single LCV generates membrane blebs during transition to a ghost cell. Images were captured every 5 min over 11 h and the playback rate of the movie is 7 frames/ s.

Supplementary Video S2: Time-lapse microscopy demonstrating the conversion of an LCV to a ghost cell, followed by the complete degradation of the cell. Images were captured every 5 min over 24 h and the playback rate of the movie is 5 frames/ s.

Supplementary Video S3: Explosive lysis of an LCV. The membrane can be seen deforming prior to lysis. Images were captured every 5 min over 19 h and the playback rate of the movie is 8 frames/ s.
